# Medical Association of Central New York

**Published:** 1890-06

**Authors:** 


### Society Meetings.

Medical Association of Central New York.—Upon the earnest request of many members the annual meeting of the Medical Association of Central New York is postponed from May 29th to Tuesday, June 10, 1890. The regular day conflicts with the date of the National meetings. Announcement of programme, place of meeting, and important business to be transacted will be issued about June 1st. By order of W. J. Herriman, President; Edw. B. Angell, Secretary.

